# Precuneal hyperperfusion in patients with attention-deficit/hyperactivity disorder-comorbid nociplastic pain

**DOI:** 10.3389/fphar.2024.1480546

**Published:** 2024-10-17

**Authors:** Miwako Takahashi, Satoshi Kasahara, Tsutomu Soma, Taito Morita, Naoko Sato, Ko Matsudaira, Shin-Ichi Niwa, Toshimitsu Momose

**Affiliations:** ^1^ Department of Molecular Imaging and Theranostics, Institute for Quantum Medical Science, National Institutes for Quantum Science and Technology, Chiba, Japan; ^2^ Department of Anesthesiology and Pain Relief Center, The University of Tokyo Hospital, Tokyo, Japan; ^3^ Department of Pain Medicine, Fukushima Medical University School of Medicine, Fukushima, Japan; ^4^ Software Development Department, PDRadiopharma Inc., Tokyo, Japan; ^5^ Nursing Department, The University of Tokyo Hospital, Tokyo, Japan; ^6^ Department of Psychiatry, Aizu Medical Center, Fukushima Medical University, Fukushima, Japan; ^7^ Institute of Engineering Innovation, School of Engineering, The University of Tokyo, Tokyo, Japan

**Keywords:** nociplastic pain, attention-deficit/hyperactivity disorder, methylphenidate, cerebral blood flow, single-photon emission computed tomography

## Abstract

**Introduction:**

Nociplastic pain (NP), a third category of chronic pain, offers a framework for elucidating its pathophysiology and treatment strategies. One of the characteristics of NP is comorbidity of symptoms other than pain, such as psychological and cognitive problems; therefore, these can be clues to understanding NP. Recently, we reported several cases with comorbid symptoms of attention-deficit/hyperactivity disorder (ADHD). Notably, ADHD medications, including methylphenidate (MP) and atomoxetine, improved chronic pain as well as the symptoms of ADHD. However, in clinical settings, identifying comorbid ADHD in patients with chronic pain is challenging, and underlying mechanisms have not been elucidated. To explore the common characteristics of brain function in patients with ADHD-comorbid NP, we identified brain regions where cerebral blood flow (CBF) distributions changed between pre- and post-treatment using single-photon emission computed tomography (SPECT). Additionally, we examined brain regions where CBF values correlated with clinical scores.

**Methods:**

We retrospectively studied 65 patients (mean age 53 ± 14 years; 30 males and 35 females) with ADHD-comorbid NP who underwent CBF-SPECT before and after ADHD medication initiation. Clinical scores included the clinical global impression severity (CGI-S), pain numerical rating scale, hospital anxiety and depression scale, pain catastrophizing scale, and Conners’ adult ADHD rating scale-self report scores. Voxel-based statistical methods were used to compare pre- and post-treatment CBF-SPECT images to identify significant differences and investigate brain regions correlated with clinical scores.

**Results:**

The CBF was higher in the precuneus, insular gyrus, and thalamus before treatment than after treatment (paired t-test, cluster-definition p < 0.001, cluster-extent threshold p < 0.05, with family-wise error [FWE] correction). The hyperperfusion in the precuneus was positively correlated with the CGI-S score and significantly reduced after treatment with MP (paired t-test, cluster-definition p < 0.005, cluster-extent threshold p < 0.05, with FWE correction).

**Conclusion:**

The finding of precuneal hyperperfusion may provide insight into the mechanisms of NP and help identify patients who would benefit most from ADHD medications.

## 1 Introduction

Nociplastic pain (NP) has been proposed as a third category of pain, distinct from nociceptive or neuropathic pain. This is because the mechanisms of NP are remained to be eludicated and may overlap those of nociceptive or neuropathic pain but cannot be explained by these two mechanisms alone (Kosek et al., 2016; Fitzcharles et al., 2021; Kosek et al., 2021). Although the symptoms of NP are diverse, it is characterized by comorbidities with various neurological symptoms other than pain, such as sleep disturbances, fatigue, and cognitive dysfunction, including attention and memory impairments (Kosek et al., 2021). Identifying the presence of comorbidities may be helpful in diagnosing and treating patients with NP, especially when effective pharmacotherapy for these comorbidities is available.

Among the various co-existing psychological issues or cognitive impairments, symptoms of attention-deficit/hyperactivity disorder (ADHD) are frequently identified in patients with fibromyalgia (van Rensburg et al., 2018; Kasahara et al., 2021a; Pallanti et al., 2021), chronic low back pain (Kasahara et al., 2021b; Ibrahim and Hefny, 2022; Kasahara et al., 2022a; Kasahara et al. 2023a; Kasahara et al. 2023c), and idiopathic orofacial pain (Kasahara et al., 2022b; Kasahara et al. 2023b; Kasahara et al. 2023c), which are representative diseases associated NP. Moreover, 72.5% of patients with NP were shown to have comorbid ADHD (Kasahara et al., 2020). Although ADHD is characterized by inattention and/or hyperactivity-impulsivity (American psychiatric Association, 2013), predominantly observed in childhood, the symptoms persist into adulthood in approximately 40%–70% of cases (Biederman et al., 2010; Ebejer et al., 2012). ADHD medications typically include dopamine stimulants, such as methylphenidate (MP), and noradrenaline transporter inhibitors, such as atomoxetine (ATX), suggesting that dopaminergic and noradrenergic nervous system dysfunctions underpin ADHD (Stahl, 2021).

Under physiological conditions, dopamine and noradrenaline are also crucial for pain modulation, contributing to endogenous analgesia via brain networks and descending pathways (Wood, 2008; Suto et al., 2023). A mouse model with ADHD induced by selective dopaminergic neuron impairment exhibited lower pain thresholds and reduced spinal descending analgesia (Bouchatta et al., 2022; Meseguer-Beltrán et al., 2023; Sifeddine et al., 2023). In an epidemiological study of the general population, an increase in ADHD symptoms was significantly associated with a higher risk of pain, indicating a common underlying mechanism between ADHD and chronic pain (Stickley et al., 2016). In addition to ADHD, individuals with NP frequently present with other psychiatric disorders, such as autism, insomnia, depression, and anxiety, all of which can significantly impact the quality of life (Wiwe Lipsker et al., 2021). Notably, ADHD medications have been shown to improve symptoms, including chronic pain (Vorobeychik and Acquadro, 2008; Kasahara et al., 2017; Wiwe Lipsker et al., 2018; Kasahara et al., 2020; Kasahara et al. 2022b; Kasahara et al. 2023c; Kasahara et al. 2023a; Kasahara et al. 2023d; Kasahara et al. 2023e; Kasahara et al. 2023b; Zain et al., 2023; Kasahara et al., 2024). Therefore, detecting ADHD comorbidity in patients with NP could be crucial for effective treatment, especially in those who are refractory to other treatments.

We previously reported in case studies that changes in cerebral blood flow (CBF) using single-photon emission computed tomography (SPECT) after treatment occurred in patients with ADHD-comorbid NP who showed improvements with ADHD medication use (Kasahara et al., 2023c; Kasahara et al. 2023a; Kasahara et al. 2023e; Kasahara et al., 2024). Before treatment, there was a relative CBF increase in the posterior cingulate gyrus, precuneus, and insular gyrus, which was alleviated after treatment, and hypoperfusion in the frontal lobe was improved. These changes may reflect the pathophysiological basis of ADHD-comorbid NP.

In this study, we aimed to 1) identify brain regions of statistically significant CBF changes in a large number of patients who improved with ADHD medication use and 2) identify brain regions that correlate with clinical scores, such as ADHD severity and pain intensity scores. Using CBF-SPECT to assess brain function, we provided objective insights into the mechanisms underlying ADHD-comorbid NP, which may be useful for diagnosing and treating patients with NP.

## 2 Materials and methods

### 2.1 Participants

We retrospectively identified consecutive patients who were referred to a psychiatrist (S.K.) from 2016 to 2023, where they were routinely evaluated using the numerical rating scale (NRS) (Jensen and Karoly, 2011), hospital anxiety and depression scale (HADS) (Zigmond and Snaith, 1983), pain catastrophizing scale (PCS) (Sullivan et al., 1995), and Conners’ adult ADHD rating scale self-report (CAARS-S) (Conners et al., 1999) at treatment initiation and during CBF-SPECT sessions. The inclusion criteria were as follows: 1) a diagnosis of chronic pain considered to be ADHD-comorbid NP, 2) at least two CBF-SPECT sessions including CBF-SPECT performed before ADHD medication use, 3) no history of brain surgery, 4) the absence of other neurological disorders or psychosis, and 5) age ≥18 years at the first visit.

This retrospective study was conducted in accordance with the Helsinki Declaration and its later amendments and approved by our institutional review board (IRB) (approval number 20–011). The IRB waived informed consent because of the retrospective nature of the study. However, the participants were informed through our institute websites of the possibility to withdraw from or refuse to participate in the study. Patient data were anonymized to protect their privacy.

### 2.2 Assessment of NP severity and post-treatment improvement

NP severity and NP improvement with ADHD medication use were determined using the clinical global impression severity (CGI-S) and clinical global impression improvement (CGI-I) scores, respectively (Busner and Targum, 2007). The CGI-S and CGI-I scores were determined by evaluating the degree of interference with daily life due to NP and cognitive impairments, such as anxiety, depression, insomnia, attention impairment, and sensory sensitivity; the degree of improvement in these scores was also evaluated. The CGI-S score ranges from 1 to 7, where 1 = normal, 2 = borderline illness, 3 = mildly ill, 4 = moderately ill, 5 = markedly ill, 6 = severely ill, and 7 = extremely ill. The CGI-I score was used to assess NP relative to the baseline condition as follows: 1 = very much improved, 2 = much improved, 3 = minimally improved, 4 = no change, 5 = minimally worse, 6 = much worse, and 7 = very much worse.

### 2.3 Clinical scores

Subjective pain was evaluated using the NRS, an 11-point pain rating scale, with 0 indicating no pain and 10 indicating the highest pain. The minimum, maximum, and mean NRS scores were assessed (Jensen and Karoly, 2011). The minimum clinically important difference (MCID) in the NRS score for chronic pain is 1 point, and a decrease of 2 points or more is considered “much better” (Salaffi et al., 2004).

Anxiety and depression were assessed using the HADS-anxiety (HADS-A) and HADS-depression (HADS-D) scores (Zigmond and Snaith, 1983). A HADS score ≥11 is rated as clinically significant for adjustment disorder or major depressive disorder (Kugaya et al., 1998), with 1.5 being the MCID (Puhan et al., 2008).

Pain-related catastrophizing thoughts were assessed using the PCS (Sullivan et al., 1995). A PCS score of ≥30 represents clinically relevant levels and corresponds to the 75th percentile or higher in the distribution of patients with chronic pain (Sullivan, 2009); the MCID score is 6.48 (Suzuki et al., 2020). Catastrophizing thoughts reflect the psychological condition of patients with chronic pain, which further heightens pain and disability (Sullivan et al., 2001).

Subjective ADHD symptoms were assessed using the long version of CAARS-S (Conners et al., 1999). The long version of CAARS consists of 66 questions, and its key feature is that it allows the severity of a patient’s ADHD symptoms to be quantified as a T-score, indicating where they fall within the population distribution for their age group. CAARS is the most widely used self-administered adult ADHD rating scale in controlled clinical studies. The ADHD index, which is the most important overall subscale score among the eight subscale scores of CAARS-S, indicates the degree to which the symptoms of patients with ADHD require treatment. Therefore, we used the ADHD index as a representative score for statistical comparisons. ADHD was diagnosed according to the Diagnostic and Statistical Manual of Mental Disorders, fifth edition, using a structured diagnostic interview for ADHD in adults (Kooij et al., 2019) and considering information about patient symptoms from all family members.

### 2.4 Medication algorithm

**FIGURE 1:**
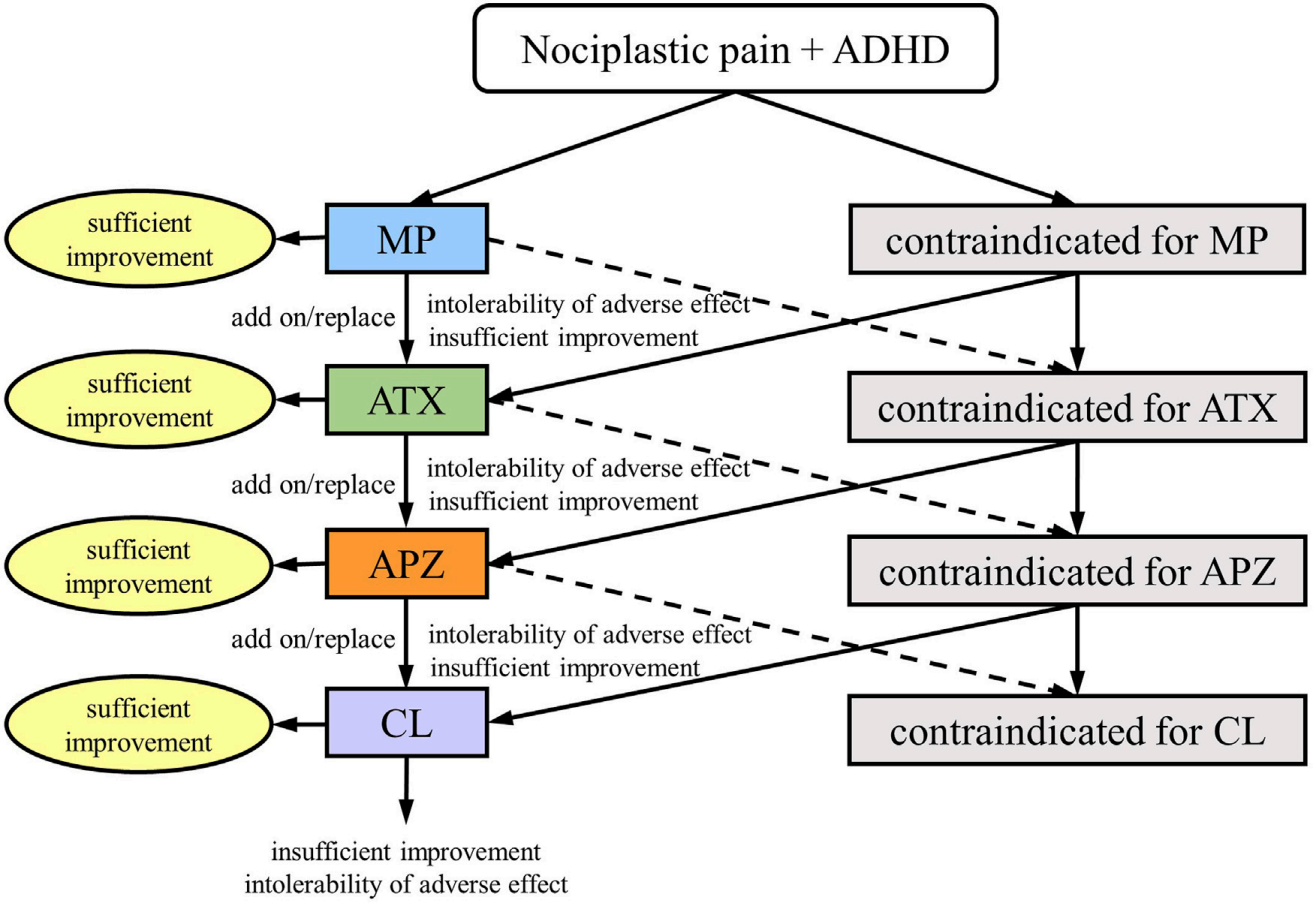
Medication algorithm for nociplastic pain in patients with attention-deficit/hyperactivity disorder (ADHD); MP, methylphenidate; ATX, atomoxetine; APZ, aripiprazole; CL, clonidine. (This figure is a modified excerpt from the original paper by Kasahara et al. (Kasahara S, Takahashi K, Matsudaira K, Sato N, Fukuda KI, Toyofuku A, Yoshikawa T, Kato Y, Niwa SI, Uchida K. Diagnosis and treatment of intractable idiopathic orofacial pain with attention-deficit/hyperactivity disorder. Sci Rep 2023; 13:1,678. DOI: 10.1038/s41598-023-28931-3, CC BY 4.0).

Treatments were administered according to the medication algorithm proposed by S.K. (Figure 1) (Kasahara et al., 2023d). Briefly, MP was used as the drug of choice in patients without contraindications (Stahl, 2021). MP is started at 18 mg/day, and the dosage is adjusted to the appropriate level for the patient in seven increments: 27 mg/day, 36 mg/day, 45 mg/day, 54 mg/day, 63 mg/day, and 72 mg/day, while monitoring for side effects such as headaches and loss of appetite. In case of insufficient MP efficacy or intolerable side effects, the medication was changed to a combination of MP and ATX or ATX alone. ATX is started at 40 mg/day and gradually increased to the standard dosage of 80–120 mg/day, adjusting to the appropriate dose for the patient while monitoring for side effects such as constipation and loss of appetite. Similarly, based on the efficacy and side effects, the medications were combined or changed successively to aripiprazole (APZ) and clonidine (CL). APZ, a partial dopamine D2 receptor agonist, is referred to as a dopamine system stabilizer because it acts as an inhibitor or activator when dopamine neurotransmission is excessive or low, respectively. APZ is started at 3 mg/day and gradually increased up to a maximum of 30 mg/day, adjusting to the appropriate dose for the patient while monitoring for extrapyramidal symptoms as a potential side effect. APZ ameliorates symptoms of chronic pain (Kasahara et al., 2011), idiopathic orofacial pain (Tu et al., 2019; Watanabe et al., 2022; Kasahara et al., 2023d), and ADHD (Ghanizadeh, 2013). CL, a noradrenaline alpha 2 receptor agonist, is effective in ADHD treatment (Stahl, 2021). CL is started at 150 µg/day and gradually increased up to a maximum of 450 µg/day, adjusting to the appropriate dose for the patient while monitoring for dry mouth and low blood pressure. For patients with severe depression accompanied by psychomotor retardation, antidepressants were used, and for those with bipolar disorder, anticonvulsants/mood stabilizers were administered. Sleep medications were also used for insomnia. Other than that, no medications outside of the treatment algorithm in Figure 1 were used. After adjusting the dose to enhance efficacy and confirming the absence of severe side effects for at least 2 months, the overall treatment effect was evaluated using the NRS, HADS, PCS, and CGI-C scores.

### 2.5 CBF-SPECT acquisition

Commercially available ^99m^Tc-labeled ethyl cysteinate dimer (ECD; PDRadiopharma Inc. Chuo-ku, Japan) was used for CBF-SPECT imaging. Patients rested in the supine position with their eyes closed in a quiet SPECT room. A 740-MBq (20 mCi) dose of ECD was injected intravenously; approximately 5 min later, a 30-min scan was performed using a triple-head SPECT system (GCA-9300R; Cannon Medical Systems, Otawara, Japan) equipped with a high-resolution fan-beam collimator, which permitted a spatial resolution of 7.2 mm full-width at half-maximum (FWHM). The SPECT images were reconstructed using filtered backprojection with Butterworth and Ramp filters. Data were collected in a 128 × 128 × 89 matrix with a voxel size of 1.72 × 1.72 × 3.44 mm.

The images were prepared for axial, coronal, and sagittal views with a rainbow color after co-registration between the pre- and post-treatment CBF-SPECT images. All CBF-SPECT images were visually reviewed by two nuclear medicine experts (M.T. and T.M.), and characteristic findings were investigated.

### 2.6 Statistical analysis

#### 2.6.1 CBF-SPECT voxel-based statistical analysis

First, the pre- and post-treatment SPECT images were realigned and averaged for each patient. The averaged images were anatomically standardized using a segmentation-based spatial normalization method implemented in Statistical Parametric Mapping 12 (Wellcome Institute for Cognitive Neurology, London, United Kingdom; RRID:SCR_002592). Subsequently, the realignment and spatial normalization parameters obtained through the abovementioned image processes were applied to the individual images to transform them into the Montreal Neurological Institute coordinate system. Finally, the transformed images were smoothed using a three-dimensional isotropic Gaussian kernel with a FWHM of 10 mm.

The voxel values of the CBF-SPECT images were standardized by dividing them by the mean number of counts per voxel in the cerebellum. To clarify the changes in regional CBF between pre- and post-treatment, a paired *t*-test was conducted for patients with post-treatment CGI-I scores of 1–3. Additionally, we focused on psychostimulant-induced CBF changes and performed a paired *t*-test for patients who showed improvement with MP use. Brain regions where CBF values were associated with clinical scores, such as CGI-S, pain NRS, ADHD index, HADS, and PCS scores, were investigated using regression analysis. Statistically related regions were detected using the cluster-extent-based thresholding method (Woo et al., 2014), which comprised two steps. First, clusters were defined by contiguous voxels above a cluster-definition threshold *p*-value. In this study, we set the cluster-definition *p*-value to <0.001 and <0.005 for the paired *t*-test and regression analysis, respectively. Second, the cluster size (number of voxels) was statistically tested under the null hypothesis. We set a cluster-extent threshold *p*-value of <0.05 with family-wise error correction.

#### 2.6.2 Clinical score statistical analysis

Clinical scores, including CGI-S scores as well as the maximum, mean, and minimum scores of NRS, HADS-A, HADS-D, PCS, and ADHD index, were compared before and after treatment using paired *t*-tests and Wilcoxon signed-rank tests for parametric and non-parametric numerical data, respectively. The *p*-value was initially calculated without correction for multiple comparisons, and then the significance threshold was set at *p* < 0.00625, specifically 0.05 divided by 8 (Bonferroni correction for a total of eight comparisons). Statistical analyses were performed using IBM SPSS Statistics for Windows, version 26 (IBM Corp., Armonk, NY, United States). Summary statistics were expressed as mean (standard deviation) for continuous variables and frequency (percentage) for categorical variables.

## 3 Results

Overall, 65 patients (mean age, 53 ± 14 years; 30 males and 35 females) were included in this study. The chief complaints in these patients were pain in three or more sites (n = 19, 29%), low back pain (n = 16, 25%), orofacial pain (n = 14, 22%), and pain in other areas (lower limb pain [n = 4]; abdominal pain [n = 3]; anal, perineal, and genital pain [n = 3]; shoulder and upper limb pain [n = 2]; pelvic pain [n = 2]; cervical pain [n = 1]; and thoracic pain [n = 1]). After treatment, 61 patients had CGI-I scores ranging from 1 to 3. The mean dose for patients receiving MP alone (n = 17) was 57.2 ± 20.6 mg/day. For patients receiving MP in combination with ATX or APZ (n = 20), the mean doses were as follows: 60.9 ± 20.2 mg/day (MP) and 101.9 ± 25.1 mg/day (ATX); 61.2 ± 11.7 mg/day (MP) and 5.3 ± 7.2 mg/day (APZ). For patients receiving ATX alone (n = 15), the mean dose was 72.7 ± 44.8 mg/day. For patients receiving ATX in combination with APZ or CL (n = 4), the mean doses were as follows: 75.0 ± 63.6 mg/day (ATX) and 2.5 ± 0.7 mg/day (APZ); 120.0 mg/day (ATX) and 375 ± 100 μg/day (CL). The mean dose was 6.0 ± 3.5 mg/day in patients receiving APZ alone (n = 2). After treatment, the number of patients with clinically significant HADS-A, HADS-D, and PCS scores decreased from 24 (37%) to 15 (23%), 31 (48%) to 17 (26%), and 39 (60%) to 21 (32%), respectively. The number of patients with ADHD index T-score ≥65 decreased from 20 (31%) to 12 (18%) after treatment.

The CGI-S score, as well as the maximum and mean NRS, HADS-A, HADS-D, and PCS scores, significantly decreased after treatment. The mean ADHD index T-score decreased from 61.7 to 56.9, although this change was not statistically significant. Based on CAARS, T-scores of ≥65, 60–65, and ≤60 are clinically significant, borderline, and within the normal range, respectively. Thus, this study suggests that after treatment, the mean T-scores fell within the normal range. Details of the clinical scores are presented in Table 1, and the number of patients at each stage of CGI-S is shown in Table 2. Compared to pre-treatment, post-treatment results showed a shift in the number of patients toward less severe stages, indicating an overall improvement in the severity.

**TABLE 1:** Details of the clinical scores and statistical comparisons.

	Pre-treatment	Post-treatment	p-value
CGI-S	5.2 ± 1.0	3.6 ± 1.1	p < 0.001*
Pain NRS			
Maximum	5.9 ± 2.4	4.6 ± 2.8	p < 0.001*
Average	4.9 ± 2.2	3.6 ± 2.7	p < 0.001*
Minimum	2.7 ± 2.4	2.3 ± 2.7	p = 0.028
HADS			
Anxiety	9.8 ± 5.2	6.9 ± 5.5	p = 0.005*
Depression	9.8 ± 5.4	8.0 ± 5.8	p = 0.005*
PCS	30.7 ± 13.2	22.7 ± 15.0	p < 0.001*
ADHD Index (CAARS-S subscale)			
T-score	61.7 ± 12.3	56.9 ± 14.0	p = 0.007

CGI-S, clinical global impression severity; NRS, numerical rating scale for pain; HADS, hospital anxiety and depression scale; PCS, pain catastrophizing scale.

* Significance level was set at *p* < 0.00625 after Bonferroni correction for multiple comparisons, with a total of eight comparisons.

**TABLE 2:** The number of patients at each stage of clinical global impression severity.

CGI-S stage	1	2	3	4	5	6	7
Pre-treatment	0	0	3	14	18	28	2
Post-treatment	2	3	27	20	10	3	0

CGI, clinical global impression severity. The scores were determined by evaluating the degree of interference with daily life due to NP and cognitive impairments, ranging from 1 to 7, where 1 = normal, 2 = borderline illness, 3 = mildly ill, 4 = moderately ill, 5 = markedly ill, 6 = severely ill, and 7 = extremely ill.

**FIGURE 2:**
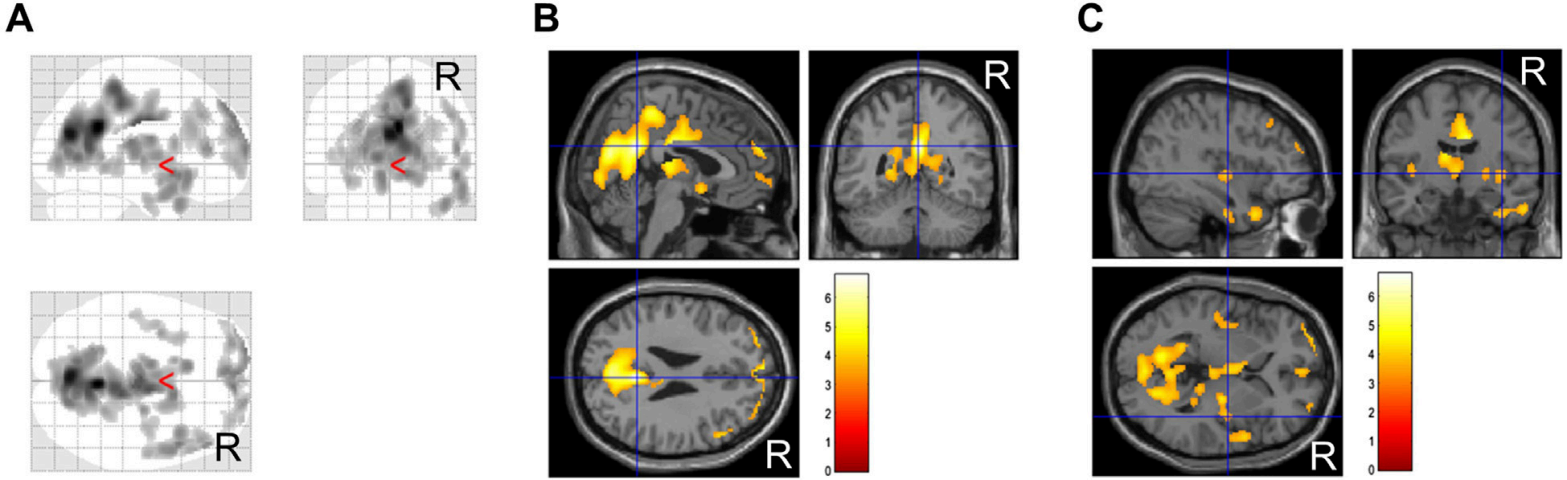
Significantly higher cerebral blood flow regions in pre-treatment images than in post-treatment images. The regions with significant changes are projected in three directions in gray **(A)** and colored (t-value) scales with cross-bars indicating the precuneus **(B)** and right insular cortex **(C)**. The medial thalamus is also identified as a higher cerebral blood flow region **(C)**. Cluster-extent *p* < 0.05 with family-wise error correction. R, patients’ right side.

The duration of pre- and post-treatment CBF-SPECT was 32 ± 18 months, ranging from 7 to 74 months. Regarding the comparison of CBF-SPECT images before and after treatment in 61 patients with CGI-I scores of 1–3, the CBF was significantly higher in the precuneus, insular gyrus, and medial thalamus in pre-treatment CBF-SPECT images (Figure 2). No brain region showed significantly lower CBF in pre-treatment CBF-SPECT images and higher or lower CBF in post-treatment images.

**FIGURE 3:**
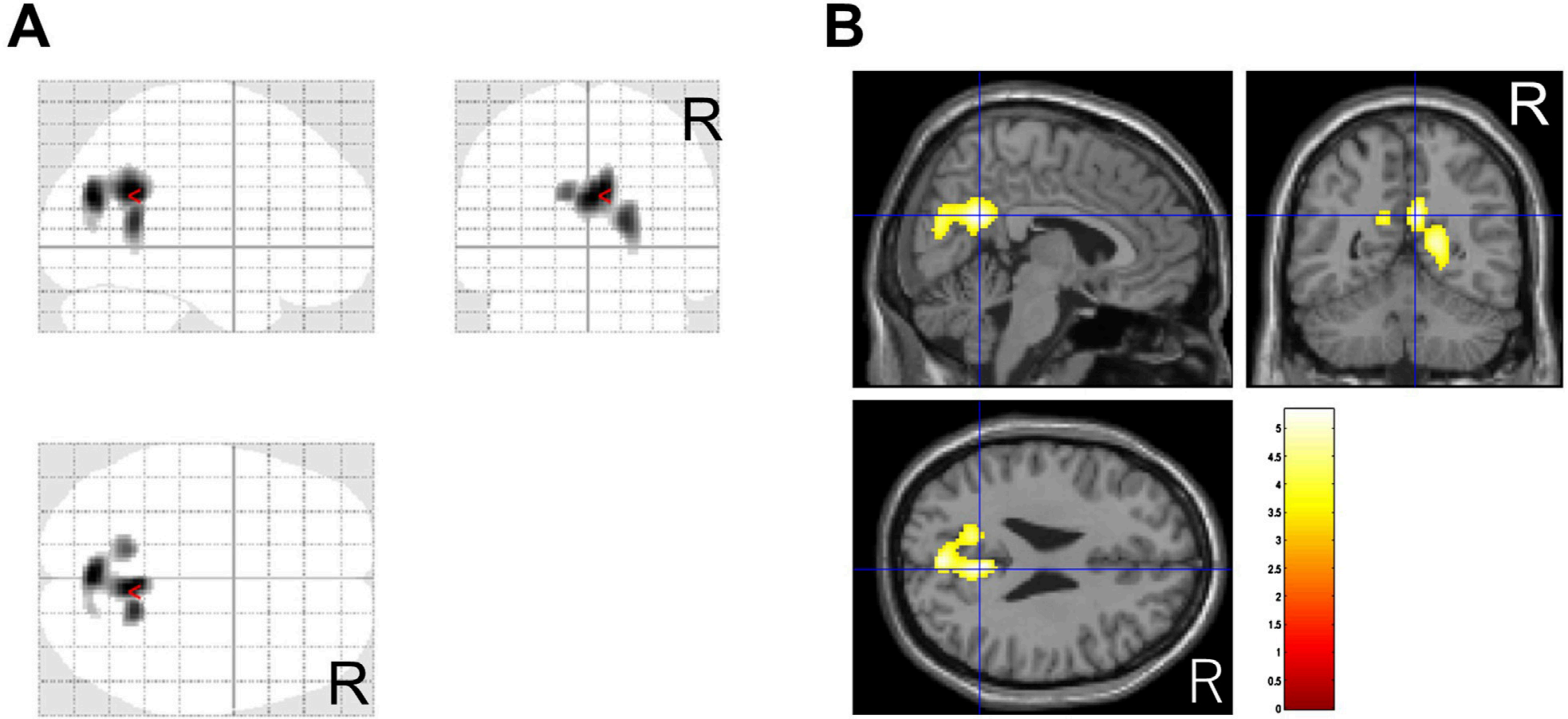
Significantly higher cerebral blood flow regions before methylphenidate treatment than after treatment. The significant regions are projected in three directions in gray **(A)** and colored (t-value) scales with a cross-bar indicating the precuneus **(B)**. Cluster-extent *p* < 0.05 with family-wise error correction. R, patients’ right side.

In this study, 37 patients were treated with MP or a combination of MP and other drug(s), and 36 of them had CGI-C scores of 1–3 after treatment. Before and after comparisons of CBF-SPECT images in these 36 MP-treated patients showed significantly higher precuneal perfusion in pre-treatment CBF-SPECT images (Figure 3).

**FIGURE 4:**
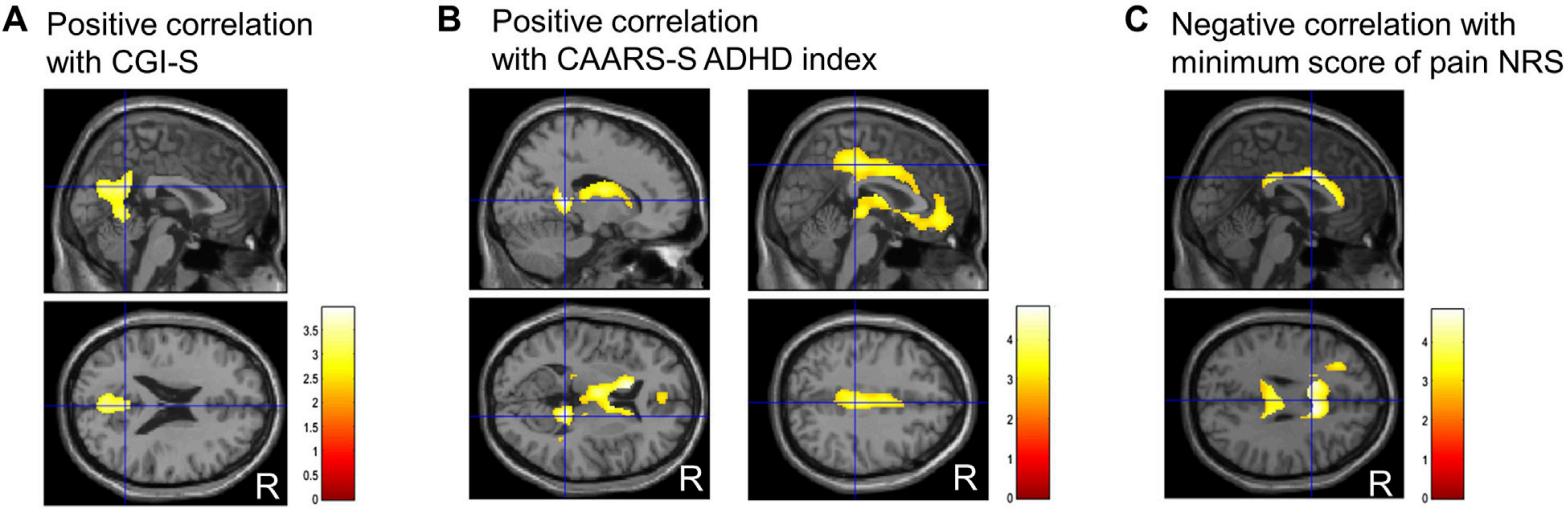
Representative images of significantly correlated regions with clinical scores. Clinical global impressions severity (CGI-S) score positively correlates with the precuneus **(A)**. ADHD index, a subscale of Conners’ adult ADHD rating scale self-report (CAARS-S), positively correlates with the isthmus of the posterior cingulate gyrus, anterior and middle cingulate gyrus, medial thalamus, and caudate head **(B)**. The minimum numerical rating scale (NRS) score for pain negatively correlates with the narrow region along the corpus callosum in the cingulate gyrus **(C)**. Cluster-extent p < 0.05 with family-wise error correction. R, patients’ right side; ADHD, attention-deficit/hyperactivity disorder.

Regarding the correlation analysis of regional CBF values with clinical scores, CBF was positively correlated with the CGI-S score in the precuneus (Figure 4A). Furthermore, CBF was positively correlated with the ADHD index in the isthmus of the posterior cingulate gyrus, anterior and middle cingulate gyri, medial thalamus, and caudate head (Figure 4B). A negative correlation was observed between CBF and minimum NRS score in regions along the corpus callosum in the cingulate gyrus (Figure 4C). No other clinical scores were significantly correlated with regional CBF values.

**FIGURE 5:**
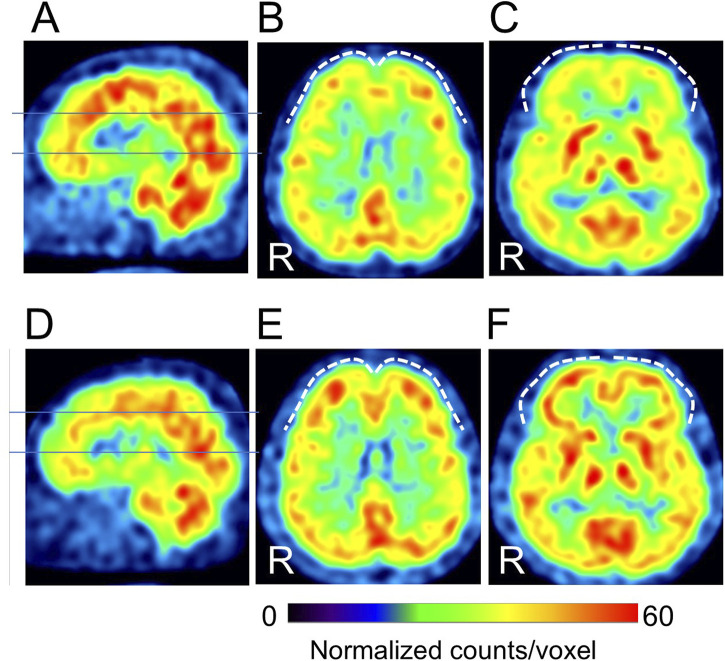
A typical example of our cases with hypofrontal perfusion (dashed curves) in cerebral blood flow single-photon emission computed tomographic images (SPECT) before treatment **(A–C)**, which is improved after treatment **(D–F)** (patient # 8). The lines in A and D indicate the axial levels of images **(B, C, E, F)**. The voxel values are normalized by the mean number of counts per voxel in the cerebellum being 50; the color ranges from 0 to 60. R, patients’ right side. This case had no psychiatric comorbidities based on the Diagnostic and Statistical Manual of Mental Disorders, fifth edition. At the time of the pre-treatment SPECT, the patient was taking 100 mg/day of tramadol hydrochloride, and at the time of the post-treatment SPECT, the medications were 25 mg/day of atomoxetine, 10 mg/day of nitrazepam, and 0.25 mg/day of triazolam.

**FIGURE 6:**
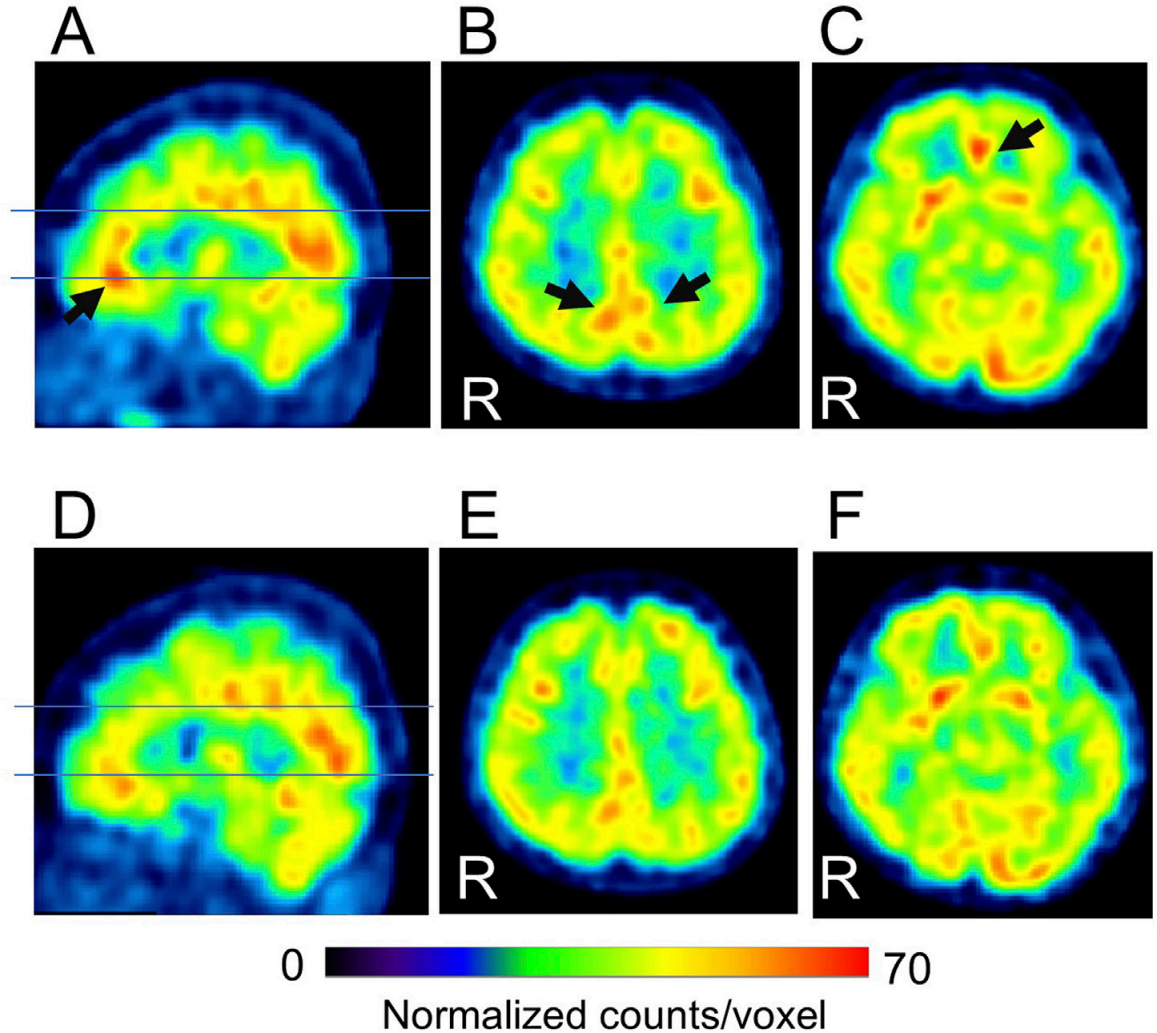
Representative images of cerebral blood flow single-photon emission tomography (SPECT) with typical findings that the subgenual anterior cingulate arrow in **(A, C)** and precuneus arrows in **(B)** show relative hyperperfusion before treatment **(A–C)**, which decreases after treatment **(D–F)** (patient # 45). The lines in A and D indicate the axial levels of images **(B, C, E, F)**. The voxel values are normalized by the mean number of counts per voxel in the cerebellum being 50; the color ranges from 0 to 70. R, patients’ right side. This case had no psychiatric comorbidities based on the Diagnostic and Statistical Manual of Mental Disorders, fifth edition. At the time of the pre-treatment SPECT, the patient was not taking any medications. At the time of the post-treatment SPECT, the medications were 10 mg/day of atomoxetine, 225 mg/day of venlafaxine, and 5 mg/day of zolpidem tartrate.

**FIGURE 7:**
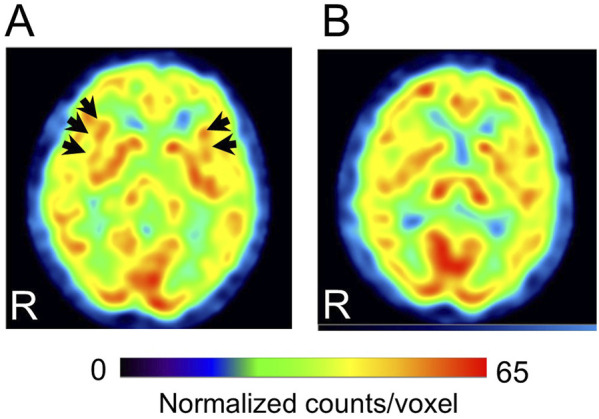
Representative images of cerebral blood flow single-photon emission computed tomography (SPECT) with a typical finding that the insular gyri show relative hyperperfusion before treatment **(A)**, which decreases after treatment **(B)** (patient # 32). Arrows in A indicate the bilateral insular gyri. The voxel values are normalized by the mean number of counts per voxel in the cerebellum being 50; the color ranges from 0 to 65. R, patients’ right side. This case had bipolar disorder as a psychiatric comorbidity based on the Diagnostic and Statistical Manual of Mental Disorders, fifth edition. At the time of the pre-treatment SPECT, the medications were 3 mg/day of etizolam, 1 mg/day of flunitrazepam, and 5 mg of zolpidem tartrate. At the time of the post-treatment SPECT, the medications were 120 mg/day of atomoxetine, 800 mg/day of sodium valproate, 25 mg/day of lamotrigine, 50 mg/day of trazodone, and 1 mg/day of flunitrazepam.

Cases with representative CBF-SPECT findings are shown in Figures 5–7. A patient in his 50s complained of low back pain before treatment and had relatively decreased frontal lobe CBF on CBF-SPECT images (Figures 5A–C). One year after ATX treatment, his symptoms improved, and CBF in the frontal lobe increased (Figures 5D–F). Figure 6 shows CBF-SPECT images of a patient in her 40s who presented with headache and orofacial pain before treatment. Pre-treatment CBF-SPECT (Figures 6A–C) showed that the CBF values in the subgenual anterior cingulate and precuneus were relatively higher than those in the surrounding frontal and parietal cortices. Two years after treatment with a dopamine system stabilizer, the symptoms improved, and the CBF in these regions decreased (Figures 6D–F). Figure 7 shows the CBF-SPECT images of a patient in her 50s with shoulder and upper limb pain before treatment. Her symptoms improved after ATX treatment, and her clinical condition remained stable 5 years later. Pre-treatment CBF-SPECT images showed relatively higher CBF in the insular gyrus (Figure 7A), which decreased in post-treatment images (Figure 7B).

## 4 Discussion

This study revealed two main findings. First, in patients with ADHD-comorbid NP, pre-treatment CBF was relatively higher in the precuneus, insular gyrus, medial thalamus, and caudate head than in other brain regions. Furthermore, in selected patients with post-MP treatment improvements, precuneal CBF was higher before treatment. Interestingly, precuneal CBF values positively correlated with CGI-S scores, reflecting NP severity. Second, regarding the relationship between regional CBF values and other clinical scores, ADHD symptoms were positively correlated with CBF in the anterior and middle cingulate gyri, medial thalamus, and caudate head, and the minimum NRS score was negatively correlated with CBF in the narrow region along the corpus callosum in the cingulate gyrus.

Several reports have suggested that pain perception and regulation are performed in multiple brain regions through networks. Processes from the perception of physical pain stimuli to higher-order cognition are conceptualized as being performed by different levels of cortical networks (Garcia-Larrea and Peyron, 2013). In the first-order network, painful stimuli generated by nociception are transmitted from the dorsal horn of the spinal cord to the thalamus through spinothalamic projections. In the second-order network, pain is transmitted from the thalamus to the anterior cingulate and insular gyri where it is perceived as an unpleasant sensation. These brain regions of the second-order network constitute the so-called pain matrix, part of which was identified as having higher CBF in this study; these findings were consistent with those of previous studies showing high perfusion or metabolism in the insular gyrus in response to unpleasant pain stimuli (Schreckenberger et al., 2005; Boly et al., 2008). Furthermore, pain-related information is relayed through the insular gyrus to higher cortical areas, such as the frontal and parietal lobes. Physical pain stimuli are emotionally recognized as discomfort caused by the pain matrix (Borsook et al., 2016). However, integrating this perception of unpleasant pain stimuli with individual knowledge and experience requires a higher level of networking, which enables it to be perceived as a personal experience. This can be considered a third-order network, although specific findings and patterns have not yet been elucidated (Garcia-Larrea and Peyron, 2013).

The increased precuneal CBF observed in this study may constitute part of the third-order network in ADHD-comorbid NP. The precuneus, which is the posterior part of the default mode network (DMN), is responsible for maintaining awareness and attention to the environment via DMN activation at rest (Fiset et al., 1999; Laureys et al., 1999; Raichle et al., 2001). Conversely, the brain regions responsible for the performance of cognitive tasks primarily comprise the prefrontal cortex, known as the central executive network (CEN), which is inactive at rest. Persistent DMN activity during task performance may interfere with CEN activity; therefore, switching DMN and CEN activities is crucial for task execution (Sridharan et al., 2008). Functional magnetic resonance imaging studies have also suggested that psychostimulant use suppresses DMN activity in children with ADHD, restoring brain activity patterns similar to those of typically developing children and improving behavioral performance (Peterson et al., 2009; Querne et al., 2017). Our finding that pre-treatment precuneal hyperperfusion was associated with pain severity and was suppressed after ADHD medication use, resulting in the improvement of pain and ADHD symptoms, was consistent with the abovementioned previous study findings. Usui et al. reported hyperperfusion in the posterior cingulate gyrus and precuneus in patients with fibromyalgia, a representative NP-associated disorder. Notably, the gabapentin (an analog of the neurotransmitter γ-aminobutyric acid [GABA]) treatment-resistant group had higher CBF values in these regions than the treatment-responsive group (Usui et al., 2010), suggesting that a different nervous system than the GABAergic system was involved in these gabapentin treatment-refractory patients. Both previous and present study results suggest that the hyperactive status of the precuneus contributes to ADHD-comorbid NP and effectively responds to ADHD medications.

We also found a positive correlation between ADHD symptoms and a relative increase in CBF in the cingulate gyrus, which is consistent with the findings of previous studies showing that ADHD is associated with hyperactivity of the cingulate gyrus (Vieira de Melo et al., 2018). A study using a mouse model with ADHD induced by dopaminergic nerve damage demonstrated that hyperactivity of the anterior cingulate gyrus (ACG) results in electrical hyperactivity of the spinal dorsal horn nerve via ACG-insular gyrus fiber communication, resulting in hyperalgesia (Bouchatta et al., 2022). Another study using a spontaneously hypertensive rat model of ADHD showed that decreased pain threshold was associated with high noradrenaline levels in the dorsal horn of the spinal cord and low alpha2-adrenoceptor levels in postsynaptic sites (Suto et al., 2023). This study showed ACG hyperactivity in the resting state, which may contribute to the descending analgesic dysfunction and ADHD symptoms. Another significant area was found in the narrow region along the corpus callosum in the cingulate gyrus that correlated negatively with the minimum NRS. This region is known to have a high density of receptors, as observed in a diprenorphine receptor PET (Vogt et al., 1995), and may be associated with pain suppression. However, there are insufficient previous studies or basic data reports on pain regulation in the cingulate gyrus.

To the best of our knowledge, this is the first study to identify brain regions associated with both pain and ADHD symptom improvements following ADHD medication use. The regions with significant improvements were the precuneus, insular gyrus, and cingulate gyrus. Notably, the precuneal CBF was correlated with NP severity and showed significant changes after MP treatment. The insular and cingulate gyri are parts of the pain matrix, whereas the precuneus is a crucial component of the DMN; therefore, it is likely related to ADHD symptoms. Considering that more than 80% of ADHD symptoms are missed in general psychiatric practice (Ginsberg et al., 2014) and that patients with NP usually consult anesthesiologists or orthopedic surgeons, the CBF-SPECT findings would enhance the suspicion of ADHD underlying chronic pain and the need to consult to a pain management specialist.

This study had some limitations. First, we did not use a non-ADHD group in this study; thus, we could not separately investigate whether ADHD medication use altered CBF in ADHD- or pain-related regions. Second, this study evaluated and compared the pre-treatment characteristics of CBF-SPECT images with those of post-treatment CBF-SPECT images as a reference. Therefore, future studies should clarify these characteristics and enhance their clinical applicability by comparing them with those of patients with NP without ADHD or normal individuals.

In conclusion, this study demonstrated a positive correlation between regional precuneal CBF and NP severity, and the CBF reduced after ADHD medication use, with pain improvement. Therefore, our study proposes the CBF findings as clues for identifying patients who would benefit from ADHD medications.

## Data Availability

The authors may provide anonymized data in compliance with the ethical guidelines and privacy regulations. A data transfer agreement must be executed and approved by the Institutional Review Board (IRB) of our institution before data can be provided. Requests for access, including clear research purposes, should be directed to the corresponding author. Data will be shared in accordance with the approval conditions set by the IRB.

## Abbreviations

NP, nociplastic pain; ADHD, attention-deficit/hyperactivity disorder; CBF, cerebral blood flow; SPECT, single-photon emission computed tomography; CGI-S, clinical global impression severity; MP, methylphenidate; ATX, atomoxetine; NRS, numerical rating scale; HADS, hospital anxiety and depression scale; PCS, pain catastrophizing scale; CAARS-S, Conners’ adult ADHD rating scale self-report; IRB, institutional review board; CGI-I, clinical global impression improvement; MCID, minimum clinically important difference; HADS-A, HADS-anxiety; HADS-D, HADS-depression; APZ, aripiprazole; CL, clonidine; ECD, ethyl cysteinate dimer; FWHM, full-width at half-maximum; DMN, default mode network; CEN, central executive network; GABA, γ-aminobutyric acid; ACG, anterior cingulate gyrus.
